# Dissecting the Structure–Activity Relationship of Galectin–Ligand Interactions

**DOI:** 10.3390/ijms19020392

**Published:** 2018-01-29

**Authors:** Yi-Chen Chan, Hsien-Ya Lin, Zhijay Tu, Yen-Hsi Kuo, Shang-Te Danny Hsu, Chun-Hung Lin

**Affiliations:** 1 Institute of Biological Chemistry, Academia Sinica, No. 128, Academia Road Section 2, Nan-Kang, Taipei 11529, Taiwan; cyc1786@yahoo.com (Y.-C.C.); pipis_lsy@hotmail.com (H.-Y.L.); s42304@yahoo.com.tw (Z.T.); yenkuo1@gmail.com (Y.-H.K.); sthsu@gate.sinica.edu.tw (S.-T.D.H.); 2 Taiwan International Graduate Program (TIGP), Sustainable Chemical Science and Technology (SCST), Academia Sinica, Taipei 11529, Taiwan; 3 Department of Applied Chemistry, National Chiao Tung University, Hsinchu 300, Taiwan; 4 Institute of Biochemical Sciences, National Taiwan University, Taipei 10617, Taiwan; 5 Department of Chemistry, National Taiwan University, Taipei 10617, Taiwan; 6 The Genomics Research Center, Academia Sinica, Taipei 11529, Taiwan

**Keywords:** binding interaction, galectin, inhibitor, isothermal titration calorimetry, NMR (Nuclear Magnetic Resonance), thiodigalactoside, X-ray crystallography

## Abstract

Galectins are β-galactoside-binding proteins. As carbohydrate-binding proteins, they participate in intracellular trafficking, cell adhesion, and cell–cell signaling. Accumulating evidence indicates that they play a pivotal role in numerous physiological and pathological activities, such as the regulation on cancer progression, inflammation, immune response, and bacterial and viral infections. Galectins have drawn much attention as targets for therapeutic interventions. Several molecules have been developed as galectin inhibitors. In particular, TD139, a thiodigalactoside derivative, is currently examined in clinical trials for the treatment of idiopathic pulmonary fibrosis. Herein, we provide an in-depth review on the development of galectin inhibitors, aiming at the dissection of the structure–activity relationship to demonstrate how inhibitors interact with galectin(s). We especially integrate the structural information established by X-ray crystallography with several biophysical methods to offer, not only in-depth understanding at the molecular level, but also insights to tackle the existing challenges.

## 1. Introduction

Lectins are carbohydrate-binding proteins that recognize a diversity of glycan structures existing in numerous glycoconjugates. As unique members of the lectin family, galectins are small, cytosolic proteins that bind β-galactosides. Albeit the lack of a signal sequence required for protein secretion, galectins usually exist both intra- and extracellularly and were thus proposed to follow a nonclassical non-Golgi/ER secretion pathway [1]. The β-galactoside-binding feature is attributed to the evolutionally conserved carbohydrate recognition domain (CRD) where the glycan binding takes place [2,3]. So far, 15 members of the galectin family have been identified in mammalians and were named in accordance with their sequential order of discovery. These galectins (abbreviated as GALs) are categorized into three groups according to the organization of their CRD [4]. Prototype galectins (including GAL-1, -2, -5, -7, -10, -11, -13, -14, and -15) form homodimers in solution via a noncovalent interaction through their single CRD, while tandem-repeat galectins (GAL-4, -6, -8, -9, and -12) comprise two distinct CRDs at their N- and C-termini that are tethered by a linker of variable length. In a chimera structure, GAL-3 is the only member that has a CRD at the C-terminus and a short non-lectin peptide motif (Gly–Pro–Tyr-rich domain) at the N-terminus. GAL-3 can self-oligomerize into a pentamer or other forms to form an adhesive network when bridging carbohydrates on the cell surface through multivalent interactions [5,6].

**Figure 1:**
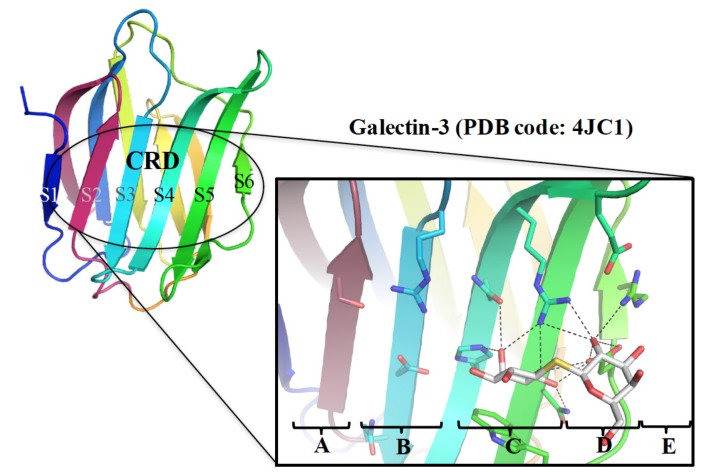
Galectin CRD contains five subsites (A–E; GAL-3 is shown here for instance) where thiodigalactosides bind, interacting with subsites C and D in a similar manner to lactose or *N*-acetylactosamine.

The CRD consists of 130 amino acid residues that fold into two antiparallel β-sheets of six (S1–S6) and five (F1–F5) strands, jointly forming a β-sheet sandwich structure and named S-sheets and F-sheets, respectively. The S1–S6 β-strands make up a concave surface to which specific glycans (up to the length of tetrasaccharides) are bound (Figure 1). To discuss the binding interactions in detail, the binding groove is further subdivided into subsites A–E, with the principle subsite C that is highly conserved among all galectin members. Subsite C harbors β-galactose, whereas the less conserved subsite D accommodates the sugar residue (e.g., glucose in lactose) next to β-galactose. In contrast, subsites A, B and E are more variable and thus specific for individual galectins. These subsites are occupied by sugars or functional groups adjacent to β-galactose.

## 2. Galectins in Cancer and Translational Medicine

Protein-carbohydrate interactions are essential for biomolecular communications within and between cells. Since galectins are capable of recognizing β-galactoside-containing glycoconjugates on the cell surface, in extracellular matrices, or in the lumen of intracellular vesicles, they play a pivotal role in apoptosis [7], cell proliferation [8], inflammation, immune response [9,10,11,12], and cell adhesion [13,14,15] and migration [8,16,17,18]. These activities, in turn, have significant roles in various diseases, such as cancer progression [5], HIV [19], autoimmune disorders, fibrosis, arthritis, obesity, cardiovascular diseases, allergies, and microbial infections [20].

Galectins are potential biomarkers in chronic or acute heart failure [21], as well as attractive targets for anticancer or anti-inflammatory agents. Cancer development is initiated when normal cells undergo neoplastic transformation that causes the dysregulation of cell growth or of regulatory mechanisms, resulting, for instance, in defective apoptosis and cell cycle alterations [5]. Several galectins are closely associated with these processes. Among them, GAL-1 and -3 are well studied for their roles in cancer progression. Previous studies demonstrated that GAL-3 is involved in processes linked to tumorigenesis, including the transformation to a malignant form, metastasis, and the increased invasiveness of tumor cells [22,23]. Particularly, GAL-3 is important for promoting cell–cell interactions and enabling cellular motility, which explains why cancer cells take advantage of GAL-3 to move and grow [24]. Furthermore, GAL-3 might also be secreted by tumor cells to sustain the microenvironment they favor, which subsequently helps them to escape from immune surveillance in two ways: (1) by inhibiting the afferent arm of the immune system, preventing the body from increasing the number of T cells in response to the tumor, and (2) by inhibiting the efferent arm, which is used to attack the tumors [22,25,26]. Accordingly, all this strongly suggests that blocking the function of GAL-3 is likely a feasible option to rescue the immune system’s ability to attack cancer cells.

**Figure 2:**
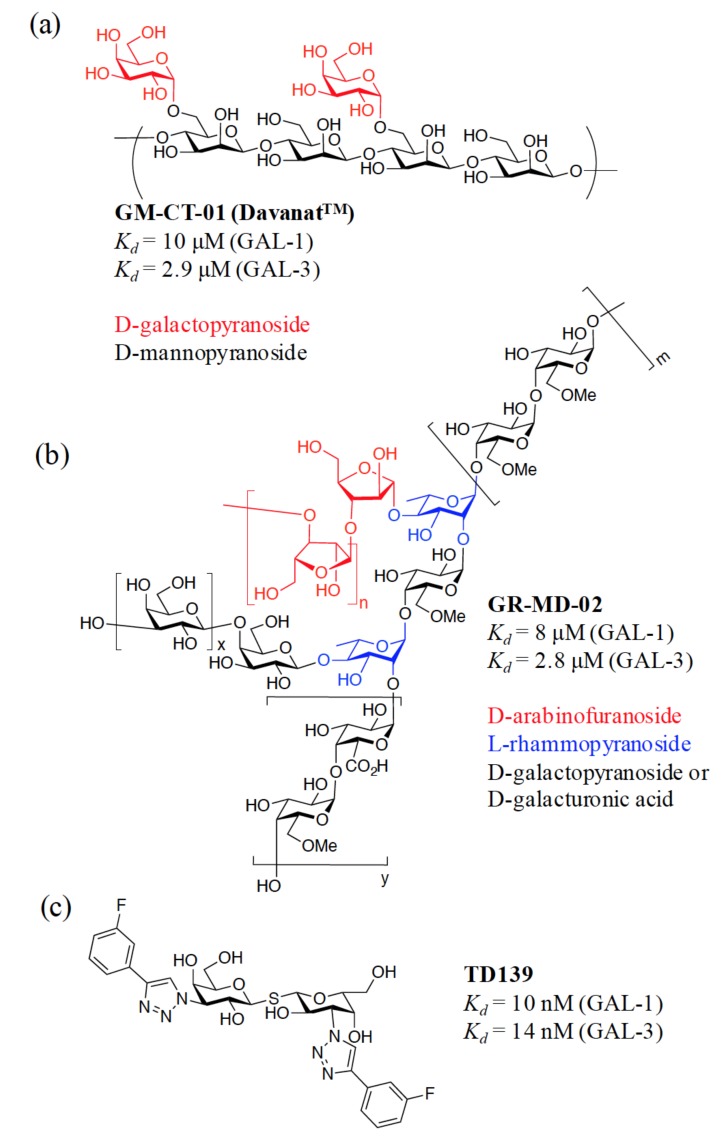
Structures of GM-CT-01, GR-MD-02, and TD139 that have been examined in clinical trials for GAL-3-related diseases.

It is a challenging task to develop selective inhibitors for GAL-3, because the desirable inhibitors have to distinguish GAL-3 from the other members. Intracellular GAL-3, for instance, displays apoptosis-suppressing activity, but extracellular GAL-3 plays the opposite role and promotes apoptosis like GAL-1, -2, and -9 [5,27,28,29]. The high similarity in the CRD structures is another issue. The differential activities of specific galectins in normal and pathological processes [30,31] also explain the urgent need to develop potent and selective inhibitors. To date, there are three different categories of GAL-3 inhibitors to attenuate cancer progression. Two of them have been advanced to clinical trials, indicating that they could be available for therapeutic intervention in the near future. Firstly, G3-C12 is a peptide (the sequence: ANTPCGPYTHDCPVKR) discovered by phage display exhibiting an excellent inhibition (*K_d_* = 72 nM) towards GAL-3 [32]. This peptide was shown to prevent the metastasis of breast cancer cells to the lung. However, several pieces of information still remain ambiguous, including its inhibition potency towards other galectins and its mode of action. Secondly, pectin is a complex polysaccharide rich in anhydrogalacturonic acid, galactose, and arabinose. This polysaccharide can bind to GAL-3 in a multivalent manner. GBC-590 (developed by Safescience, Inc., Boston, MA, USA.) is one of the modified citrus pectin derivatives [33,34]. It was shown to reduce colorectal carcinomas in Phase II trials [35]. Likewise, GCS-100 produced significant activity in Phase II clinical trials to treat patients suffering from relapsed chronic lymphocytic leukemia [36]. However, its binding to galectins has not been clearly demonstrated. Two additional polysaccharide-based multivalent inhibitors, GM-CT-01 (Davanat^TM^, formerly invented by Pro-Pharmaceuticals, Inc.) and GR-MD-02 (Figure 2a,b), both developed by Galectin Therapeutics, showed moderate affinity with GAL-3 (*K_d_* = 2.9 and 2.8 µM, respectively). GM-CT-01 is a natural galactomannan polysaccharide with an average molecular weight up to 60 kDa. Its polymannoside backbone is branched with galactose residues. GR-MD-02 is a galactoarabino-rhamnogalacturonan polysaccharide with a molecular weight of ~50 kDa. These molecules are currently examined under Phase I or Phase II clinical trials for several cancers [37,38,39]. Nonetheless, it was noted that both GM-CT-01 and GR-MD-02 display comparable inhibition of GAL-1 and -3 (*K_d_* = 10 µM and 8 µM for GAL-1, respectively, determined by NMR studies) [38,40,41,42]. Because of their high water solubility and safe features in humans, these plant polysaccharides are good drug candidates. The use of these pectins as galectin inhibitors is so far based on studies in cell culture and animal models. It could be risky to correlate the clinical efficacy of pectins to GAL-3-mediated activities. On the other hand, there is no clear and satisfying structural explanation on how these pectins bind to galectins and how their affinities for GAL-1 and -3 are linked to their therapeutic efficacy.

Furthermore, TD139 (Figure 2c) [43], which is in clinical development by the Swedish startup Galecto Biotech [44], is a small-sized, monovalent inhibitor. Despite its low affinity for GAL-2, -4N, -4C, -7, -8N, and -9N, TD139 displays potent inhibition of GAL-1 (*K_d_* = 10 nM, determined by fluorescence polarization (FP)) and GAL-3 (*K_d_* = 14 nM, also by FP) [45], exhibiting a high selectivity for GAL-1 and -3. This inhibitor has completed Phase Ib/IIa clinical trials for the treatment of Idiopathic Pulmonary Fibrosis. TD139 was generated several years after the optimization of TDG-based inhibitors started in 2004, [46,47,48,49,50,51], representing the combined efforts of chemical synthesis, X-ray crystallography, and computational modeling. Since thiodigalactoside (TDG) and TD139 are symmetric saccharides, and TD139 represents a TDG derivative bearing two identical substituents (4-fluorophenyl-triazole) at the C3- and C3′-positions of TDG, we prepared TAZTDG (an asymmetrical derivative of TD139), containing one 4-fluorophenyl-triazole at C3 to understand how the inhibition potency is established by an extra binding interaction with the introduction of an additional substituent [52]. Meanwhile, in addition to the resolved X-ray crystal structures, we also relied on the use of several biophysical methods to obtain insights about the binding interactions.

## 3. Rationale for the Design of Anti-Galectin Agents

**Figure 3:**
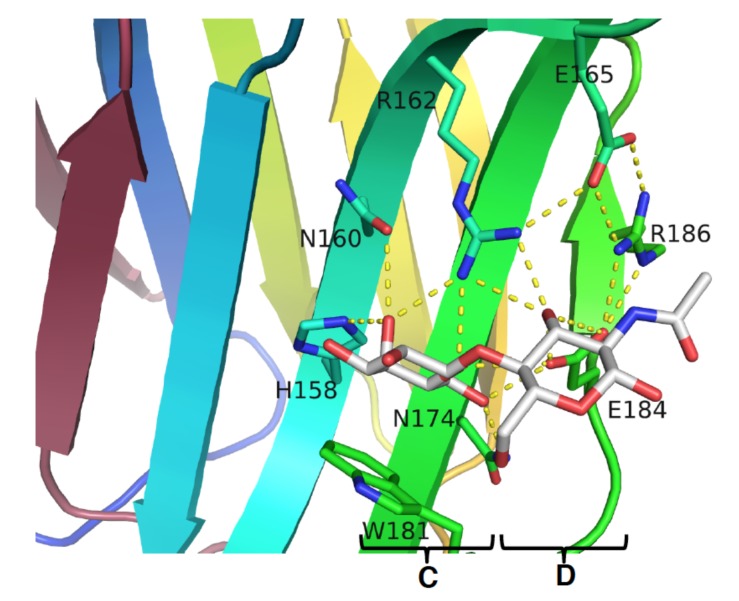
The complex structure of GAL-3/*N*-acetylactosamine (LacNAc) (PDB code: 3ZSJ). Residues in subsites C and D are highly conserved in galectins. The natural ligand LacNAc resides at subsites C and D forming several H-bonds that are shown in dotted yellow lines.

Since the majority of galectin activities is associated with their carbohydrate-binding features, the inhibition of the CRD by antagonists (or inhibitors) to compete with the natural ligand appears to be a feasible option, not only to disclose their exact functions, but also to develop molecules for therapeutic intervention. The glycotope interacting with galectin was outlined in 1986, and the first structural information came from the X-ray structure of galectin CRD in complex with lactose (PDB code: 1HLC) [53,54], which delineated the binding interactions at the molecular level. In accordance with the complex structure of GAL-3–Gal-β1,4-GlcNAc, several interactions were noticed between the galactose moiety, a series of conserved residues on S4–S6 β strands, and the loop connecting the S4 and S5 strands [55]. Taking GAL-3 as an example, the interactions included three hydrogen bonds (H-bonds) between the oxygen atom of galactose C4–OH and His158, Asn160, and Arg162, with the bond lengths of 2.8, 3.3, and 2.9 Å, respectively (Figure 3). Meanwhile, O5 was found to be H-bonded to Arg162 and Glu184, and C6–OH was found to interact with Asn174 and Glu184, so that seven H-bonds in total were observed with galactose. On the other hand, only three H-bonds were spotted on the GlcNAc moiety, between the C3–OH and the residues Arg162 and Glu184.

In addition to the H-bond network, H3, H4, and H5 of galactose were also found to interact with Trp181 via van der Waals forces. Taken together, the galactose of lactose/LacNAc is a paramount component for the recognition by galectins and is indispensable for the interactions between the CRD residues and a ligand or inhibitor.

### 3.1. Enhancement of the Binding Affinity

As the C4–OH, O5, and C6–OH of galactose are highly engaged in binding interactions, the remaining C2–OH and C3–OH of galactose do not participate in any interaction and can thus be available for modifications to gain extra binding [47,56]. Nilsson and coworkers developed the chemistry of thio-glycosides and generated a number of TDG derivatives [48,57], because TDG not only possesses a similar binding affinity as lactose/LacNAc, but also resists chemical or enzymatic hydrolysis. Like LacNAc or lactose, TDG situates at subsites C and D. Modifications of the LacNAc or TDG scaffold can introduce additional positive protein–ligands interactions (e.g., electrostatic interaction, van der Waals forces, and H-bond) at other subsites of the CRD, and this strategy has become a common approach to increase the binding affinity. Since different galectins have variations in their protein sequences at subsites A, B, and E, the introduced binding interactions at these sites would likely enhance the affinity and selectivity at the same time.

**Figure 4:**
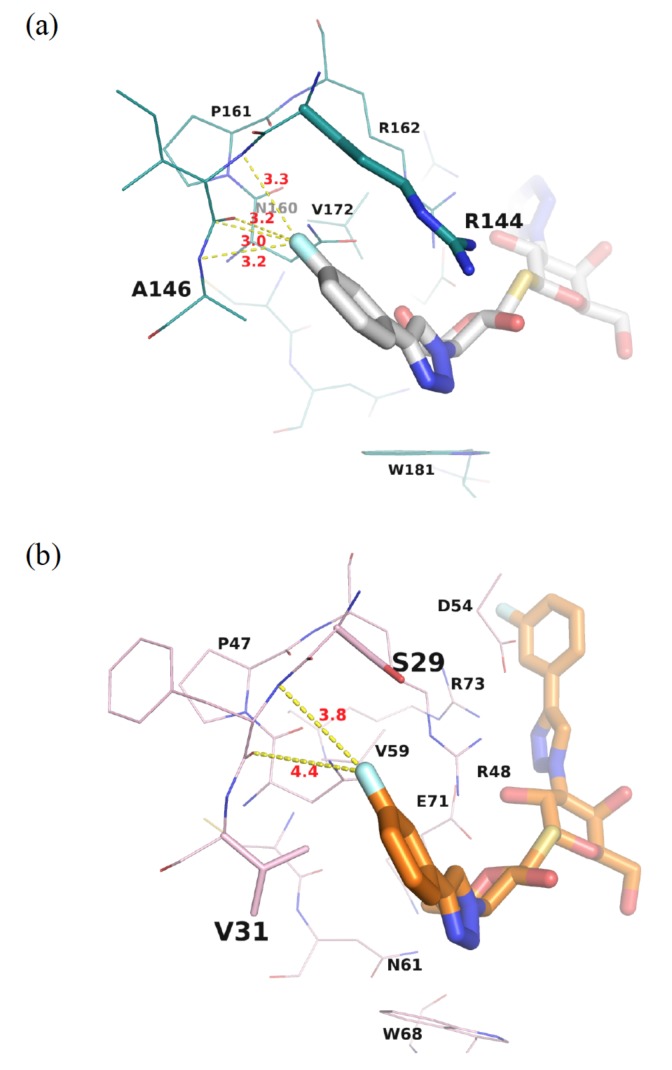
Close-up view of (**a**) GAL-3 and (**b**) GAL-1 subsite B in complex with TD139. The multiple fluorine bonds are shown as dotted yellow lines between the fluorine and the backbone peptide.

**Figure 5:**
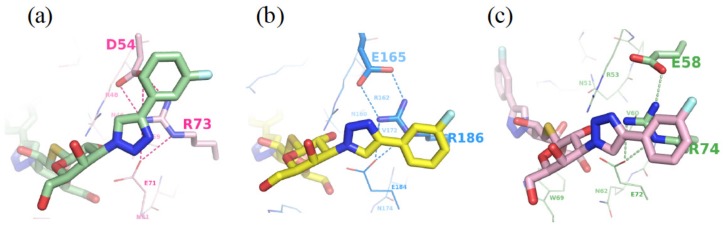
Structures of (**a**) GAL-1 (PDB code: 4Y24), (**b**) GAL-3 (5H9P), (**c**) GAL-7 (5H9Q) in complex with TD139 to display the ion-pair-π (**a**) or tandem arginine-π (**b**,**c**) interactions at subsite E. The salt bridges between Asp/Glu and Arg (in dotted lines) are essential for controlling the orientation of Asp and Arg involved in the aforementioned interactions.

TD139 is an ideal example in which the introduction of 4-fluorophenyl-triazole to the C3- and C3′ positions of TDG led to ~1000-fold higher affinity for GAL-3, as compared to TDG (*K_d_* = 0.068 and 75 µM measured by isothermal titration calorimetry (ITC) for TD139 and TDG, respectively). Several interactions were identified to contribute to this dramatic enhancement. The 4-fluorophenyl-triazole substituent was stacked between Arg144 and Ala146 at subsite B of GAL-3, forming arginine-π interactions (Figure 4a) [52]. The terminal fluorine atom of TD139 thus formed multiple, orthogonal polar interactions (namely, fluorine bondings) with the protein backbone carbonyls and backbone amide NHs, at an average distance <3.5 Å (Figure 4a), resulting in a characteristic fluorophilic microenvironment [58,59]. Meanwhile, at the other end of TD139, tandem arginine-π interactions were observed between the 4-fluorophenyl and triazole moieties and Arg186 at subsite E (Figure 5). We previously reported that the salt bridge between Asp/Glu and Arg (e.g., Arg162–Glu165–Glu184–Arg186 in GAL-3 [55,60]) is indispensable to orient the Arg residue that participates in the aforementioned arginine-π interactions.

**Figure 6:**
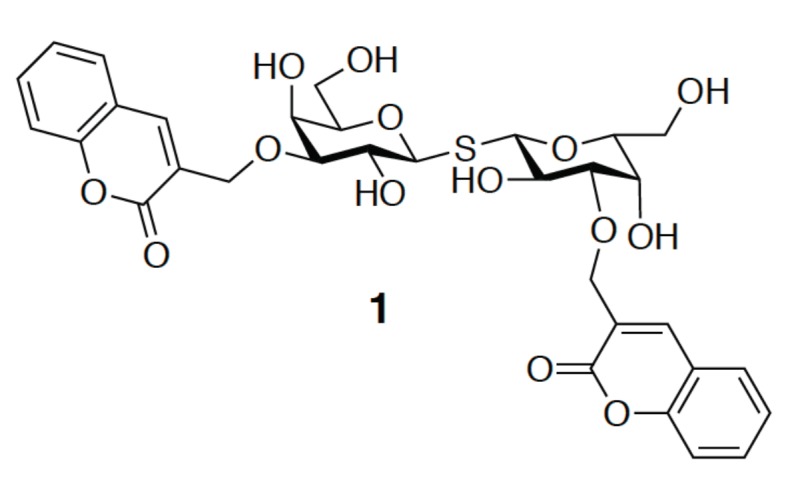
Molecular structure of di-substituted 3-*O*-coumarylmethyl thiodigalactoside (**1**).

Meanwhile, compound **1** is the analogue of TD139 containing two coumarylmethyl substituents at both positions C3 and C3′ of TDG (Figure 6) [49]. Its binding affinity for GAL-3 (*K_d_* = 91 nM) is 176-fold higher than that for GAL-1 (*K_d_* = 16 μM). It is suggested that the increment of the binding affinity is due to the two arginine-π interactions, as in TD139 where the coumarin substituents are proposed to stack on the guardinium groups of Arg144 and Arg186 in GAL-3, forming two face-to-face arene-arginine interactions. Neither of the GAL-3 mutants (R144K, R144S, R186K, or R186S) shows a prominent affinity for GAL-3. Additionally, water-mediated H-bonds were found to bridge between the coumaryl carbonyl oxygen and Lys76. Although the H-bonds contributed to the affinity, they were, however, less important than the two coumarine–arginine interactions.

### 3.2. Enhancement of the Binding Selectivity

**Figure 7:**
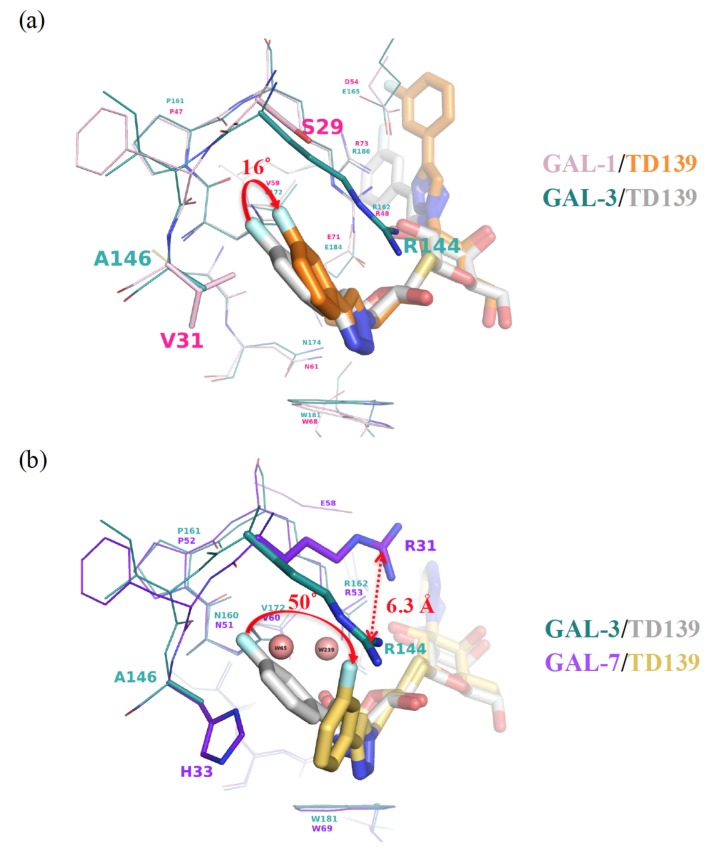
(**a**) Structural superimposition of the GAL-1 (pink)–TD139 (orange) complex and GAL-3 (deep teal)–TD139 (gray) complex. Val31 of GAL-1 is bulkier than the corresponding residue (Ala146) in GAL-3, and the 4-fluorophenyl moiety is forced to rotate approximately 16°. (**b**) When the structures of GAL-3 (deep teal)–TD139 (gray) and GAL-7 (purple blue)–TD139 (yellow orange) are superimposed, the fluorine atom of the 4-fluorophenyl moiety at GAL-7 B subsite turns approximately 50° away as compared to that in GAL-3. In addition, a large difference is observed between Arg144^GAL-3^ and Arg31^GAL-7^, that are 6.3 Å apart.

To discuss the binding specificity, we will place a special emphasis on the binding interactions at subsites B and E. When comparing GAL-1 and -3, for instance, Ser29 and Val31 at subsite B of GAL-1 correspond to Arg144 and Ala146 in GAL-3, respectively. Since Val is a little bigger than Ala, the 4-fluorophenyl moiety is forced to rotate ~16° (Figure 7a) in the GAL-1 complex, leading to the formation of only two fluorine–protein interactions, in contrast to the four interactions observed in the GAL-3 complex (Figure 4a,b). Moreover, GAL-7 contains Arg31 and His33 in similar positions as those held by Arg144 and Ala146 in GAL-3, respectively, but Arg31^GAL-7^ is remote from subsite B and is thus not close to the 4-fluorophenyl substituent of TD139. The 4-fluorophenyl moiety is found to turn ~50° away as compared to that in the GAL-3 complex (Figure 7b), likely because of the steric hindrance caused by the imidazole of His33^GAL-7^, a much bulkier residue than its counterparts Val31^GAL-1^ and Ala146^GAL-3^. Two water molecules are present in the resulting vacated space in subsite B of GAL-7 (Figure 7b). The different arrangement explains the lower affinity of TD139 for GAL-7 (*K_d_* = 38 µM) than for GAL-3 (*K_d_* = 0.068 µM, determined by ITC) [52].

**Figure 8:**
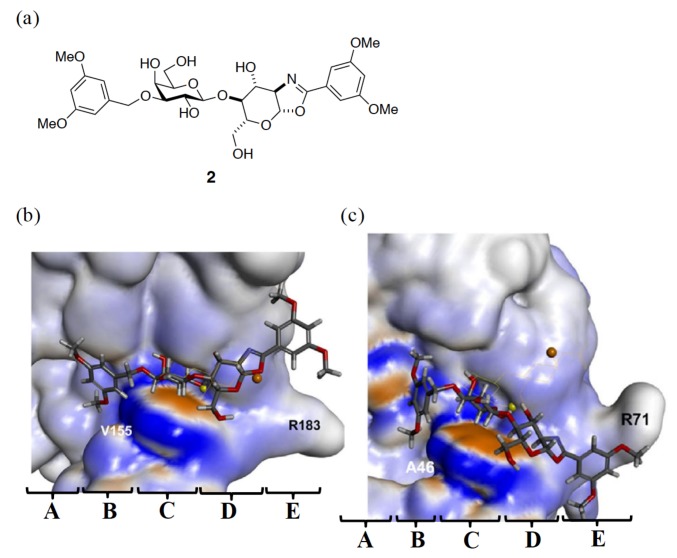
(**a**) Molecular structure of 1,2-fused (3,5-dimethoxyphenyl)-oxazoline-substituted LacNAc (compound **2**). (**b**,**c**) Postulated binding mode of **2** with GAL-3 and -7, respectively. The binding interactions were generated by the CDOCKER docking protocol using Discovery Studio version 4.1. Reprinted with permission from © 2017 Wiley-VCH Verlag GmbH & Co [61].

Furthermore, Arg183^GAL-3^ and Arg71^GAL-7^ adopt a different rotametric position when binding with 1,2-fused (3,5-dimethoxyphenyl)-oxazoline-substituted LacNAc (compound **2**, Figure 8a). Arg183 extends outside subsite E (Figure 8b), whereas Arg71 is curved back towards the lactosamine-binding site (Figure 8c) [61]. The conformational difference causes a 60-fold higher preference of compound **2** for GAL-3 (*K_d_* = 4.4 µM) than for GAL-7 (*K_d_* = 249 µM). In comparison with the binding in GAL-3, the conformational change in subsite E of GAL-7 is too narrow to accommodate the 3,5-dimethoxybenzyl-oxazoline moiety. The inhibitor, thus, has to take a different conformation to be situated at the same subsite.

**Figure 9:**
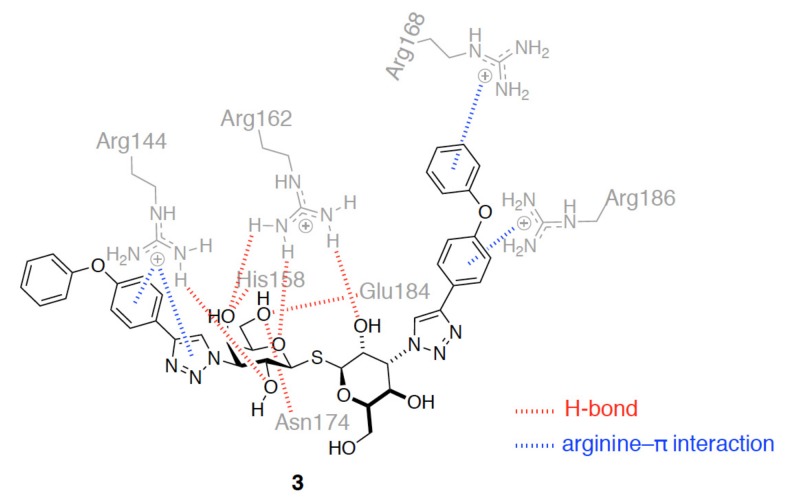
Representative binding mode of compound **3** with GAL-3 that contains H-bonds (shown in red dotted lines) and arginine-π interactions (blue dotted lines). The mentioned binding interactions were derived from the molecular docking study.

Additionally, compound **3** represents another analogue of TD139 containing diphenyl ether-linked triazole at positions C3 and C3′ of TDG (Figure 9). It shows a 230-fold higher selectivity for GAL-3 (*K_d_* = 0.36 µM) than for GAL-1 (*K_d_* = 84 µM) [62]. The augmented preference for GAL-3 was proposed to be due to the presence of three arginine-π interactions with Arg144^GAL-3^, Arg168^GAL-3^, and Arg186^GAL-3^ in subsites B and E (Figure 9). On the other hand, only one interaction (with Arg73) can be observed in GAL-1.

**Figure 10:**
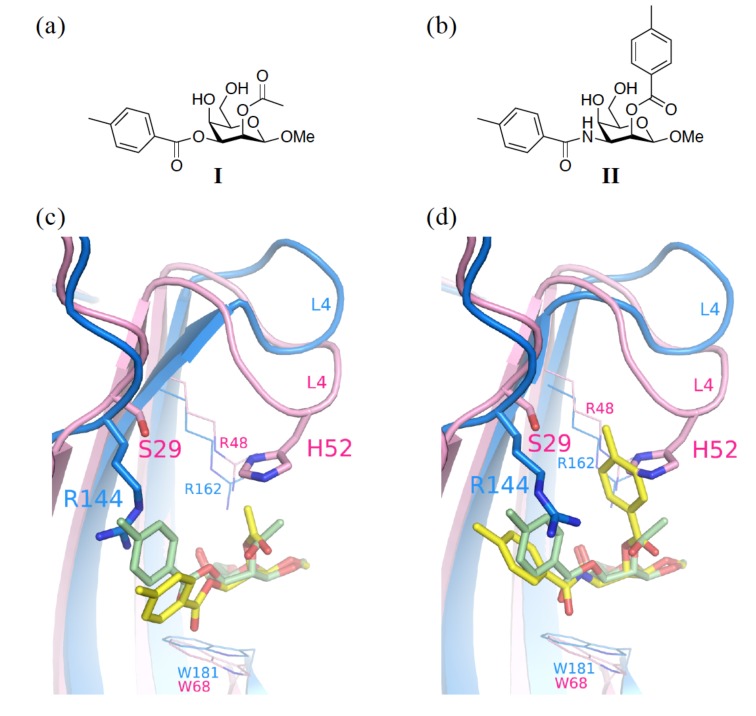
Molecular structures of the two talosides, including (**a**) methyl 2-*O*-acetyl-3-*O*-toluoyl-β-D- talopyranoside (**I**) and (**b**) methyl 3-deoxy-2-*O*-toluoyl-3-*N-*toluoyl-β-D-talopyranoside (**II**). Despite similar structures of **I** and **II**, their affinity with GAL-1 is different (*K_d_* > 4 mM and *K_d_* = 2.4 mM, respectively). (**c**) The complex structure of GAL-1 (pink)–**I** (pale green) is superimposed with that of GAL-3 (marine blue)–**I** (yellow). (**d**) The complex structure of GAL-1 (pink)–**I** (pale green) is superimposed with that of GAL-3 (marine blue)–**II** (yellow).

We previously mentioned that C2–OH of galactose is not involved in any binding interaction. It is of note that a pocket mainly created by L4 (between S4 and S5) is on the top of the galactose ring (Figure 10). One approach taking advantage of this feature is to synthesize taloside derivatives (that are the C2-epimers of galactosides; see Figure 10a,b), and introduce a substituent at C2–OH. Interestingly, His52 of GAL-1 appears to be more interactive towards the O2-substituent of talosides, whereas Arg48^GAL-1^ forms one edge of the pocket and Ser29^GAL-1^ borders the other edge (Figure 10c,d). Since Ser29^GAL-1^ is smaller than the equivalent Arg144^GAL-3^, the pocket in GAL-1 apparently has a larger space than that in GAL-3 for the close contact with the O2-substituent. Indeed, 2-*O*-Toluoyl taloside (**II**) shows higher affinity for GAL-1 than 2-*O*-acetyl taloside (**I**) and forms the proposed histidine–aromatic stacking interaction (Figure 10b,d) [63]. Unfortunately, both **I** and **II** display neither satisfying potency (mM range inhibitory potential) nor selectivity for GAL-1 and -3. With a larger, optimized O2-substituent, it is still possible to obtain a more promising result.

**Figure 11:**
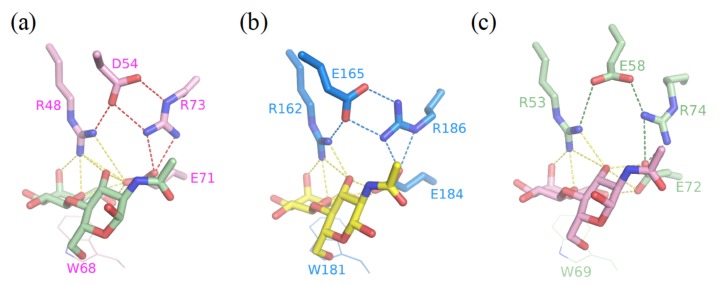
Structures of (**a**) GAL-1 (PDB ID: 1W6P), (**b**) GAL-3 (1KJL), and (**c**) GAL-7 (5GAL) in complex with LacNAc. The salt bridges between Asp/Glu and Arg in LacNAc complex structures are shown in dotted lines for GAL-1 (red), -3 (marine blue), and -7 (forest green), respectively, and all interactions related to galectin–LacNAc complexes formation are colored in yellow.

Furthermore, we previously identified an Arg-Asp–Glu-Glu-Arg salt-bridge network critical for the binding preference of Galβ1-3/4GlcNAc disaccharides for GAL-1, -3, and -7, though the salt bridge was not involved in the direct interactions with the bound ligand [55]. Notably, the salt bridge was found to be critical for the binding interaction with inhibitors. Asp54^GAL-1^, Glu165^GAL-3^, and Glu58^GAL-7^ that participate in the formation of the salt bridges (Figure 11), were found to orient the Arg residue in subsite E to interact with the 4-fluorophenyl substituent of TD139. Tandem arginine-π interactions were similar between the 4-fluorophenyl-triazole and Arg186^GAL-3^ or Arg74^GAL-7^ in the complex structures of GAL-3 and -7 with TD139. The 4-fluorophenyl-triazole moiety of TD139 was, nevertheless, found to flip over at subsite E of GAL-1. The resulting change made the aromatic substituent become close to Asp54^GAL-1^, leading to the formation of an ion-pair π-interaction, which was favored by the fluorinated arene (i.e., an electron-deficient π system) (Figure 5) [52]. Taking this difference into consideration may help to design potent inhibitors against specific galectin(s).

## 4. Tools to Characterize the Binding Interactions

X-ray crystallography is the most powerful tool to give precise insights on the arrangement of atoms of a protein–ligand complex in solid state. Nevertheless, the comprehensive understanding of protein–ligand interactions often requires collecting additional information about several pivotal aspects, such as the dynamic movement of the ligand and/or a certain part of the protein, and kinetic and thermodynamic properties of the binding process. This explains the reason why X-ray crystallography is not the only solution. To understand the binding process at a molecular level, X-ray crystal structure analysis has to be complemented with other methods to afford an insightful analysis during the stage of drug development and optimization. In the following sections, we will discuss the methods that have been employed in the development of galectin inhibitors.

### 4.1. Fluorescence Polarization

**Figure 12:**
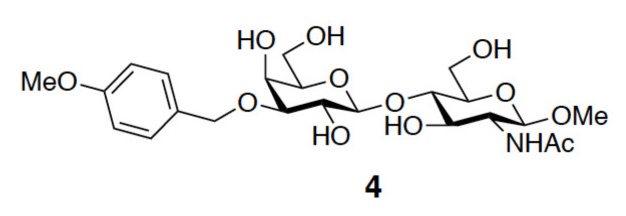
Molecular structure of 3′-*O*-*p*-methoxybenzyl ether-substituted LacNAc (compound **4**).

Fluorescence polarization (FP) allows the quantitative analysis of molecular interactions in solution by measuring the difference in the degree of polarized light of a bound and unbound fluorophore, which in turn is related to the size of the mobility and the size of the protein–probe complex. Because of its simple and rapid operation, FP has become popular for high-throughput screening in search of lead compounds via competitive binding assays. FP does not require the protein of interest to be labeled or immobilized [64,65]. In addition, the concentrations of all interacting components are known. The most beneficial credit is that the affinity of the ligands is determined when target proteins exist in solution, in a situation that mimics their biological environment. In the field of galectins, Leffler and coworkers were the first to establish an FP-based binding assay when evaluating the IC_50_ values of several 3′-*O*-benzyl ether-substituted LacNAc derivatives, in which fluorescein-tagged LacNAc served as the reference compound [46,66]. The derivative *p-*methoxy substituted benzyl ether (compound **4**, Figure 12) gave the highest inhibitory potency (IC_50_ = 13 µM) in comparison to the parent ligand, methyl glycoside of LacNAc (IC_50_ = 158 µM). FP was also applied more recently to determine the dissociation constants of a series of 3-3′ disubstituted (4-aryl-1,2,3-triazolyl)thiodigalactoside-based derivatives (e.g., TD139, Figure 2c) in relation to GAL-1, -2, -3, -4N, -4C, -7, -8N, -9N, and -9C. Overall, the disubstituted (4-aryltriazolyl) thiodigalactosides were more potent inhibitors than TDG of all the aforementioned galectins, except for GAL-8. Among these galectins, GAL-1 and -3 were strongly inhibited [45].

**Figure 13:**
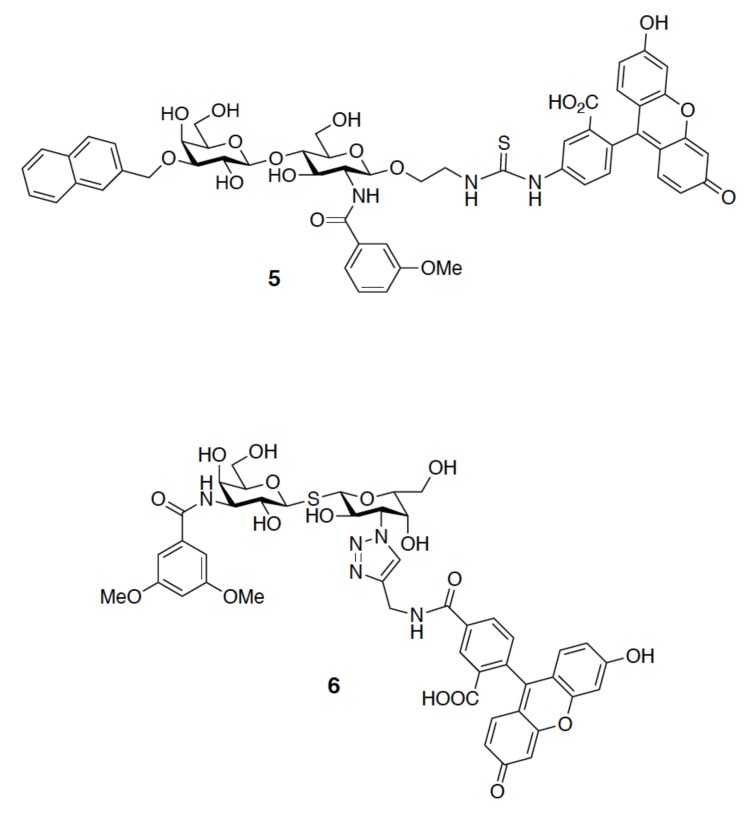
Molecular structures of 2-(fluorescein-5-ylthioureidoethyl) [3-*O*-(naphthalen-2-methyl)-β-d-galactopyranosyl]-(1→4)-2-(3-methoxybenzamido)-2-deoxy-β-d-glucopyranoside, **5** and 3,3′-dideoxy-3-[4-(fluorescein-5-yl-carbonylaminomethyl)-1H-1,2,3-triazol-1-yl]-3′-(3,5-di-methoxybenzamido)-1,1′-sulfanediyl-di-β-d-galactopyranoside, **6**.

In general, a fluorescence probe of satisfying affinity is the prerequisite of FP analysis to avoid false positives. The groups of Tamara [45] and Johann [67] obtained up to 1000-fold different *K_d_* values for GAL-1–TD139 because their fluorescent probes (**5** and **6**, respectively, Figure 13) displayed distinct affinities for GAL-1. Hence, it is noteworthy that potential lead compounds can be likely ignored at the beginning of the screening if a high-affinity probe is used. When using a probe of low affinity, it is necessary to study the target protein at high concentrations, which becomes inevitable to pick up weak-binding compounds.

### 4.2. Biolayer Interferometry

Biolayer Interferometry (BLI) is a relatively new method to measure biomolecular interactions. The proteins of interest have to be immobilized on the biosensor tip. Upon the binding with an analyte (ligand), the thickness of the biosensor tip is usually changed. BLI analyzes the optical interference pattern of a white light reflected from the immobilized surface, in comparison to an internal reference surface, thus allowing for direct real-time measurements of binding kinetics, including the rate of association (*k_on_*), the rate of dissociation (*k_off_*), and, concomitantly, the equilibrium constant (*K_d_*) [68]. BLI has become recently popular to determine *K_d_* values. Zhang et al. applied BLI for examining pectin binding of GAL-3 and comparing it with the other four methods, namely, surface plasmon resonance, FP, competitive fluorescence-linked immunosorbent assay, and cell-based hemagglutination assay [69]. Chien and coworkers measured the *K_d_* values of lactose with GAL-1, -7, -8N, -8C, and the full-length GAL-8 [70].

The lifetime of a drug–protein target complex is highly associated with the *k_off_* that is particularly important in rating the efficacy of a drug. A drug’s *k_off_* is not determined merely by its affinity, as a drug only takes effect when it is bound to the protein target [71,72,73]. Two distinct candidates may have the same level of affinity, but greatly different binding kinetics. TD139 was found to have very slow off rates (*k_off_*) in the binding with GAL-1 and -3 (10^−2^ s^−1^ and 10^−3^ s^−1^, respectively), as revealed by the BLI analysis. This result is consistent with the data measured by the ^15^N-^1^H correlation spectra. However, to our knowledge, no report has discussed how the binding kinetics of galectin inhibitors correlates with their complex half-life in solution or in a biological system.

### 4.3. Isothermal Titration Calorimetry

There have been more and more studies relying on thermodynamics measurements in drug discovery because they provides clues on the energy-driven binding interactions [74]. ITC is the ideal method to detect the heat released or absorbed during a biomolecular binding interaction down to the nanomolar range [75]. The binding affinity is determined by the change of free energy (∆G) that is in turn affected by two parameters: the change of enthalpy (∆H) and entropy (∆S) (i.e., ∆G = ∆H − T∆S). Enthalpy reflects the heat differences as a result of the binding process, whereas entropy accounts for the degree of disorder in association with the same event [76]. A number of thermodynamic analyses focus on the binding of galactose-bearing glycans with galectins. The results are consistent with the structural studies. For example, Bachhawat-Sikder et al. performed the ITC analysis for the binding of LacNAc with GAL-3 [77]. The process was enthalpically driven (∆H = −8.88 kcal/mol) with a seven times higher affinity than that of the binding of lactose, which was equivalent to 4.08 kcal/mol of favorable enthalpy change. The preferential binding was realized by the C2-acetamide of the GlcNAc moiety that interacts with Glu165 via a water molecule, in addition to the aforementioned H-bond network established by Arg162^GAL-3^ and Glu184^GAL-3^ with O3 of GlcNAc.

The binding of TDG towards GAL-1 and -3 is also an enthalpy-driven, exothermic process with a heat release of −11.6 and −9.4 kcal/mol, respectively. The introduction of one 4-flurophenyl-triazole moiety at the C3 of TDG to produce TAZTDG resulted in a significant favorable change in enthalpy, with ∆∆H_TAZTDG-TDG_ = −4.5 and −5.3 kcal/mol for GAL-1 and GAL-3, respectively. The lower energy level of TAZTDG is assumed to be a consequence of an arginine-π interaction at subsites B and E. On the other hand, the incorporation of a second 4-flurophenyl-triazole moiety did not have much effect on GAL-1 (∆∆H_TD139-TAZTDG_ = −0.6 kcal/mol), in contrast to GAL-3 (∆∆H_TD139-TAZTDG_ = −3.5 kcal/mol) [52], suggesting a differential binding at subsites B and E. This is consistent with the presence of two Arg residues at the CRD of GAL-3 (Arg144 at subsite B, Arg186 at subsite E) with the possibility of forming another arginine-π interaction. For GAL-1, however, there is only one Arg (i.e., Arg73) available, which is located at the subsite E of CRD. The additional 4-fluorophenyl-triazole moiety, does not, thus, provide much impact on the enthalpy. The effect of additional binding interactions became more obvious when comparing TD139 (*K_d_* = 68 nM) and TDG (that contains no substituent, *K_d_* = 75 μM). In addition to displaying the highest binding preference for GAL-3, TD139 possessed approximately a 1000-fold binding enhancement as compared to TDG. The great enhancement was due to a change in the free energy (ΔΔG_TD139-TDG_) of −4.3 kcal/mol, which was contributed by a huge enthalpy change (ΔΔH_TD139-TDG_ = −8.8 kcal/mol) but offset by an unfavorable entropy change (–TΔΔS_TD139-TDG_ = 4.6 kcal/mol). It is noted that the ITC method is usually suitable for inhibitors of moderate affinity (*K_d_* from sub-mM to sub-µM). When ITC is applied for the direct measurement of high-affinity inhibitors (*K_d_* of low nM and better), it always generates very steep binding curves with few data points on the slope of the curves. Since the value of *K_d_*/∆G is extracted from the shape or slope of the titration curves, the measurement often results in large errors in the *K_d_*/∆G values. Nowadays, the direct ITC measurement has been substituted by an indirect procedure in which a medium-affinity ligand is competed and displaced by the desirable high-affinity inhibitor.

Subsequent modifications to overcome the enthalpy and entropy compensation are important to ameliorate the binding affinity of a ligand. In fact, one has to be aware that experimental ∆G, ∆H, and calculated ∆S only serve as a guideline, and other molecular studies need to be integrated. ∆S is not determined solely from a separate experiment, hence any error that occurr during the measurements of ∆G and ∆H will be reflected on the entropy change [78]. Meanwhile, the presumption that in a protein–ligand binding event the net enthalpy is predominantly contributed by polar interactions while entropy is dominated by desolvation effects, does not hold in all cases [79].

### 4.4. ^19^F-NMR Spectroscopy

In the last two decades, the introduction of a fluorine atom to drug molecules has become more pronounced in medicinal chemistry [80,81] because of several key advantages, including the improvement on the metabolic stability, the regulation of the acidity and basicity, and the increase of the affinity by forming extra interactions with target proteins (e.g., H-bonds, hydrophobic, and polar interactions) [82,83]. A growing number of reports have incorporated fluorine to lead compounds in the initial stage of drug discovery. Moreover, ^19^F-NMR spectroscopy provides a rapid and sensitive way to detect the presence of fluorine-containing compounds, since biomolecules usually do not contain fluorine, and thus there is almost no background in ^19^F-NMR. This method is also useful to reveal binding interactions, because the ^19^F signals of fluorine-containing ligands would shift either upfield or downfield under different chemical environments in the bound state, in comparison with those of the free ligands. A shielded fluorine atom is thought to be in the proximity to H-bond donors within a protein structure, while a deshielded one is predominantly found in close contact with hydrophobic side chains or with the carbonyl carbon in the protein backbone [84,85].

**Figure 14:**
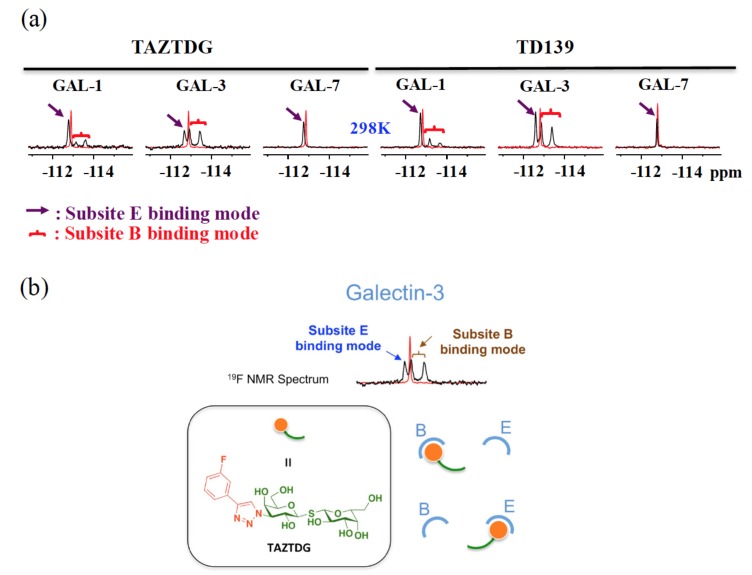
(**a**) ^19^F-NMR spectra of TAZTDG and TD139 in complex with GAL-1, -3, and -7. (**b**) Dual binding modes of the galectin inhibitor TAZTDG. On the basis of the integrated analysis of isothermal titration calorimetry, X-ray crystallography, and NMR spectroscopy, the 4-fluorophenyl-triazole moiety of TAZTDG can bind with either subsites B or E, leading to the observed dual binding modes.

We previously employed ^19^F-NMR to explore the binding features of TD139 in complex with GAL-1 and -3 [52]. The free-form ligand exhibited a single and sharp resonance at ~113 ppm as a consequence of rapid tumbling in solution (Figure 14a). Interestingly, the GAL-1 and -3 complexed with TD139 generated similar spectra that contained three distinct resonances of broader linewidths, suggesting the presence of a slow conformational interconversion of the fluorinated phenyl rings of TD139 on the NMR timescale. One unique ^19^F resonance represents a fingerprint of one binding mode of the 4-fluorophenyl moiety. Therefore, the two upfield-shifted ^19^F resonances were attributed to two different binding modes of the 4-fluorophenyl moiety that is densely packed in the subsites B of GAL-1 and -3. Meanwhile, the upfield shifts were due to the shielding effect of the multiple fluorine bonds on the fluorine atom at the subsite B. On the other hand, the most downfield-shifted resonance (δ < −113 ppm, Figure 14a) in the ^19^F spectrum was likely corresponding to the binding with the subsite E. The Arg and Asp/Glu side chains at the subsite E of both galectins established an extended π-electron surface at which the terminal fluorine was situated. The fluorine atom was deshielded, hence leading to the observed downfield shifts in the peak positions.

The asymmetrical TAZTDG in complex with GAL-1 and -3 also gave similar spectra as those of the symmetrical TD139. This indicated that the monosubstituted TAZTDG could bind with either subsites B or E in solution, in a similar manner as the di-substituted TD139, to concurrently occupy both subsites (Figure 14b). The corresponding heteronuclear ^15^N-^1^H correlation spectra further supported the dual binding modes of TAZTDG and GAL-1 or -3.

Additionally, in the ^19^F-NMR study of TD139 and TAZTDG in complex with GAL-7, only one resonance was observed in each case. This corresponded to a weak binding and, hence, fast averaging of free and bound signals, and at the same time reflected an apparent population-weighted chemical shift.

**Figure 15:**
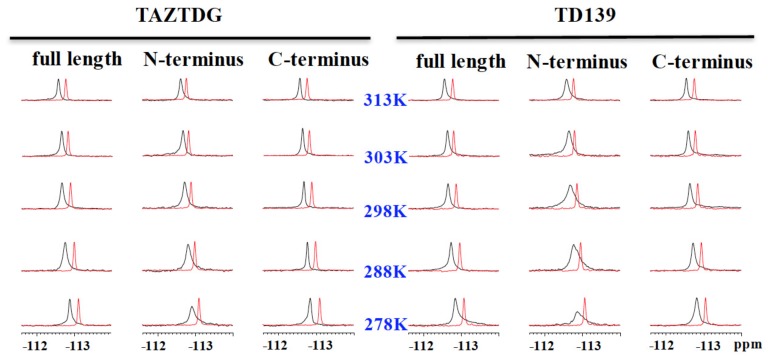
The ^19^F-NMR spectra of TAZTDG (left panel) and TD139 (right panel) in complex with GAL-8^full^, GAL-8^NCRD^, and GAL-8^CCRD^ are shown in the order of decreasing temperatures (from top to bottom). The spectra of free TAZTDG and TD139 under the same conditions are shown in red as references.

Additionally, ^19^F-NMR was also applied to examine the binding of TAZTDG and TD139 with GAL-8. This galectin is known to contain two different CRDs located at the N- and C-termini of GAL-8. We thus prepared three different forms of GAL-8, including the full length and the two individual domains (namely, N-CRD and C-CRD). Like the bound form of GAL-7 previously mentioned, the GAL-8 complexes exhibited a single broadened resonance, which reflected its low binding affinity for TAZTDG and TD139. In comparison with GAL-8^NCRD^, the signal pattern of GAL-8^CCRD^ was similar to that of GAL-8^full^ (Figure 15), implying that the C-terminal domain might be predominant in the binding of GAL-8 to TAZTDG and TD139. The observation is consistent with the *K_d_* values of TD139 (*K_d_* = 45, 88 and 14 µM for GAL-8^full^, GAL-8^NCRD^, and GAL-8^CCRD^, respectively). Additionally, the linewidths of the GAL-8^NCRD^ bound form were much broader than those of GAL-8^full^ and GAL-8^CCRD^. In spite of the lower affinity with GAL-8^NCRD^, the signals were not split even when the temperature was dropped to 278 K, which is likely explained by the slow conformational interconversion of the fluorinated phenyl rings, as mentioned previously in the study of GAL-1 and GAL-3.

There is no specific inhibitor for either N-CRD or C-CRD of GAL-8. It is known that N-CRD prefers binding to negative-charged glycans, while C-CRD binds better to neutral carbohydrates. This is in agreement with a previous study showing that 3′-sialyl LacNAc and 3′-sulfated lactose displayed higher affinity for GAL-8^NCRD^ than for GAL-8^CCRD^ [70,86,87,88]. In accordance with our ^19^F-NMR study, TD139 appeared to bind better to GAL-8^CCRD^ than to GAL-8^NCRD^, probably because the inhibitor has no charge.

## 5. Future Direction

Galectins are found primarily in the cytosol, nucleus, extracellular space or in the circulation. Their functions do not entirely take place outside the cells. Extra- and intracellular galectins often display opposite functions. Extracellular GAL-1, for instance, is responsible for promoting cancer cell proliferation, but intracellular GAL-1 plays an opposite role in inhibiting cell proliferation [89]. Another example comes from GAL-8 that was shown to play a specific role in bacterial or viral infection. After pathogens are engulfed by cells, they typically try to escape from the endosome to access nutrients in the cytosol. GAL-8 was found to recognize special glycosylation sites found within the endosomes and recruit the adapter machinery CALCOCOL2 (namely calcium-binding and coiled-coil domain-containing protein 2) that activates autophagy [90]. As a consequence, it is indispensable to develop inhibitors of high affinity to specifically target either extra- or intracellular galectins, or even selectively label their cellular locations. It is indeed a challenging issue to come up with molecules that mostly stay either outside or inside the cells.

Moreover, several selective inhibitors have been developed so far, but there are noticeable challenges remaining. One major problem is that only few galectins were examined, such as GAL-1, -3, -7, and -8. Great success has been achieved to distinguish between GAL-3 and other galectins. Most of the developed inhibitors display satisfying affinity for GAL-3 but not for weak-binding galectins (e.g., GAL-7). It is certainly a major breakthrough if the selectivity can be reversed (e.g., to develop inhibitors more selective for GAL-7 than for GAL-3).
